# The endoderm: a divergent cell lineage with many commonalities

**DOI:** 10.1242/dev.150920

**Published:** 2019-06-03

**Authors:** Sonja Nowotschin, Anna-Katerina Hadjantonakis, Kyra Campbell

**Affiliations:** 1 Developmental Biology Program, Sloan Kettering Institute, Memorial Sloan Kettering Cancer Center, New York, NY 10065, USA; 2 Bateson Centre, Firth Court, University of Sheffield, Western Bank, Sheffield, S10 2TN, UK; 3 Department of Biomedical Science, Firth Court, University of Sheffield, Western Bank, Sheffield, S10 2TN, UK

**Keywords:** Collective cell migration, Endoderm, Epithelial-to-mesenchymal transitions, Mesenchymal-to-epithelial transitions, Morphogenesis

## Abstract

The endoderm is a progenitor tissue that, in humans, gives rise to the majority of internal organs. Over the past few decades, genetic studies have identified many of the upstream signals specifying endoderm identity in different model systems, revealing them to be divergent from invertebrates to vertebrates. However, more recent studies of the cell behaviours driving endodermal morphogenesis have revealed a surprising number of shared features, including cells undergoing epithelial-to-mesenchymal transitions (EMTs), collective cell migration, and mesenchymal-to-epithelial transitions (METs). In this Review, we highlight how cross-organismal studies of endoderm morphogenesis provide a useful perspective that can move our understanding of this fascinating tissue forward.

## Abstract (precis)

**Summary:** This Review discusses the mechanisms of early endoderm morphogenesis across different model organisms, highlighting a surprising number of conserved features, including cells undergoing an epithelial-to-mesenchymal transition, collective cell migration and mesenychymal-to-epithelial transitions.

## Introduction

The endoderm is one of the earliest cell types to form in the embryo. It is the progenitor tissue that gives rise to the majority of internal organ systems of the human body, including the respiratory and gastrointestinal tracts, as well as their associated vital organs such as the thyroid, liver, pancreas, prostate and bladder. Consequently, endodermal tissues are required for many homeostatic processes, such as absorption of nutrients, gas exchange, detoxification and glucose homeostasis. This makes the proper development of the endoderm critical for many basic functions.

Until recently, the behaviour of endoderm cells during early stages of development had been relatively understudied and poorly understood. Investigating the endoderm had been hindered, in part due its inaccessibility (i.e. it being internal) and difficulties in visualizing it during normal and perturbed development, but also because it comprises just a small proportion of the bulk of cells in an embryo at any given stage. For example, the endoderm represents approximately 3.5% of all cells of the mouse embryo-proper at midgestation (Nowotschin et al., 2019). Furthermore, nascent endoderm cells are not easily morphologically distinguishable from adjacent tissues in many model organisms. Moreover, in amniotes, the squamous epithelial nature of the nascent endoderm epithelium makes gene expression hard to localize by mRNA *in situ* hybridization. However, in recent years, studies of the endoderm have been aided by the identification of robust molecular markers that identify endoderm cells, coupled with high-resolution time-lapse and, in some cases, deep-tissue imaging techniques. These approaches have yielded a wealth of new data indicating that, although endoderm organs may vary in their form and function, both within and across species, they share many mechanisms that orchestrate their earliest stages of development. These include a series of tightly coordinated and precisely timed morphogenetic processes, including epithelial-to-mesenchymal transitions (EMTs; see Glossary, Box 1), collective cell migration (see Glossary, Box 1) and mesenchymal-to-epithelial transitions (METs; see Glossary, Box 1). Increasingly, endoderm development in different organisms is being used to model these basic cellular processes (Campbell et al., 2011; Nakaya et al., 2008; Pert et al., 2015; Viotti et al., 2014b), which play key roles in the formation of many tissues and are implicated in several pathogenic events, such as cancer metastasis (Campbell et al., 2019; Campbell, 2018; Cheung and Ewald, 2016; Friedl and Gilmour, 2009; Nieto et al., 2016). The conserved features of early endoderm morphogenesis are somewhat surprising, given that although many of the upstream signals directing cells towards an endoderm identity are conserved between vertebrates, they are not conserved between invertebrates and vertebrates.

Box 1. Glossary **Blastoderm.** An epithelial layer that forms within the blastula and encloses blastocoel. Blastoderm gives rise to ectoderm, endoderm and mesoderm during gastrulation.

**Collective cell migration.** A cell migration phenomenon in which cells migrate in loosely or closely associated groups, and affect one another while doing so (Rorth, 2012).

**Diplobastic.** Animals with two germ layers.

**Egression.** Cells intercalating into an epithelium (Schöck and Perrimon, 2002).

**EMT (epithelial-mesenchymal transition).** A continuum of states characterized by loss of polarity and adhesive properties of epithelial cells and acquisition of a mesenchymal identity.

**Ingression.** Cells exiting an epithelium and moving into the body of a tissue mass (Schöck and Perrimon, 2002).

**Intercalation.** Cell neighbour exchange; for example, cells joining an epithelium or resident within an epithelium and exchanging neighbours.

**Invagination.** In-pocketing of a sheet of cells; for example, the future embryonic gut in several species.

**Mesendoderm.** Cells that can give rise to either mesoderm or endoderm, either by cell division and daughter cells having distinct fates, or in response to inductive signals from environment.

**MET (mesenchymal-epithelial transition).** Mesenchymal cells polarize and start expressing adhesion proteins to become epithelial.

**Triploblast.** Animals that derive from three definitive germ layers: ectoderm (from the Greek εκτοσ, meaning ‘outside’), mesoderm (Greek µεσοσ, ‘middle’) and endoderm (Greek ενδον, ‘inside’).

In this Review, we focus on the earliest stages of endoderm morphogenesis across different organisms, ranging from invertebrate to vertebrate models. To facilitate cross-organism comparisons, we first discuss the origin and fate of the endoderm across different organisms, as well as our understanding of the term ‘mesendoderm’ (see Glossary, Box 1). We then overview current knowledge of endoderm internalization, migration and re-epithelialization. Rather than charting evolutionary changes and similarities, we instead centre our attention on some of the principal model systems used for studying endoderm development and the key findings garnered from them, in order to provide a benchmark for cross-model studies. The gene networks that act upstream of endoderm specification have been extensively discussed elsewhere (Tremblay, 2010; Stainier, 2002; Zorn and Wells, 2007, 2009), and instead we review findings regarding the properties of endodermal cells and their behaviours.

## The origin of endoderm: where it comes from and how to define it

**Fig. 1.**
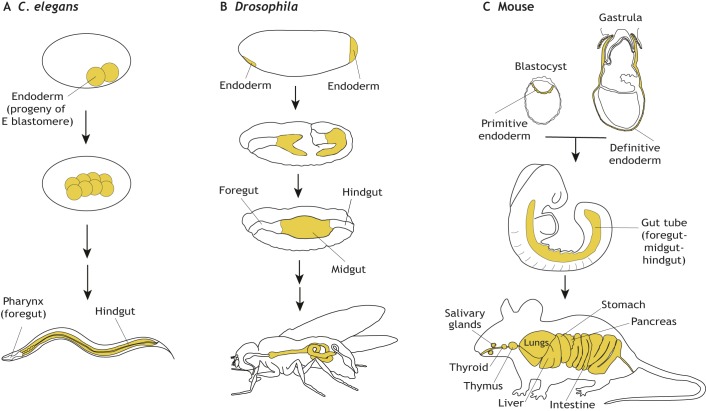
**Location of the endoderm throughout the development of worm, *Drosophila* and mouse embryos.** (A) In *C. elegans*, the endoderm is derived from the progeny of the E blastomere and gives rise to endodermal cells of the entire gut tube. (B) In *Drosophila*, endoderm forms at the anterior and posterior poles of the embryo and then invaginates to form the midgut. The foregut and hindgut of the gut tube are of ectodermal origin. (C) In mice, the entire gut tube is composed of cells of two different endodermal origins – (1) the extra-embryonic endoderm, which comprises visceral endoderm descendants of the primitive endoderm specified in the pre-implantation blastocyst; and (2) embryonic endoderm (usually referred to as definitive endoderm) – which are descendants of the epiblast, specified at gastrulation. The gut tube then gives rise to the epithelial lining of all endodermal organs of the adult mouse.

The body plans of bilatarians are triploblastic (see Glossary, Box 1), deriving from three definitive germ layers: ectoderm, endoderm and mesoderm. The mesoderm is thought to have arisen as a derivative of the endoderm around 40 million years after the emergence of endoderm and ectoderm (Stainier, 2005). This diversification of the mesodermal germ layer from the endoderm during the course of evolution has been attributed as the main driver for the increased biological diversity found in bilaterians (Technau and Scholz, 2003). During normal embryonic development, the tissue derivatives of the three germ layers become stereotypically organized, with cells of the endoderm eventually forming the epithelial lining of a gut tube that runs the length of the anterior-posterior body axis, from the mouth to the anus (Fig. 1). In invertebrates, endoderm cells are internalized during gastrulation and remain inside the organism throughout development. By contrast, in most vertebrates, with some notable exceptions such as the cephalochordate Amphioxus, endoderm cells initially move inwards during gastrulation, but then emerge on the surface of the embryo-proper where they comprise a sheet of cells. They are then later re-internalized to form the gut tube and its derivatives (Tremblay, 2010; Zorn and Wells, 2009).

**Fig. 2.**
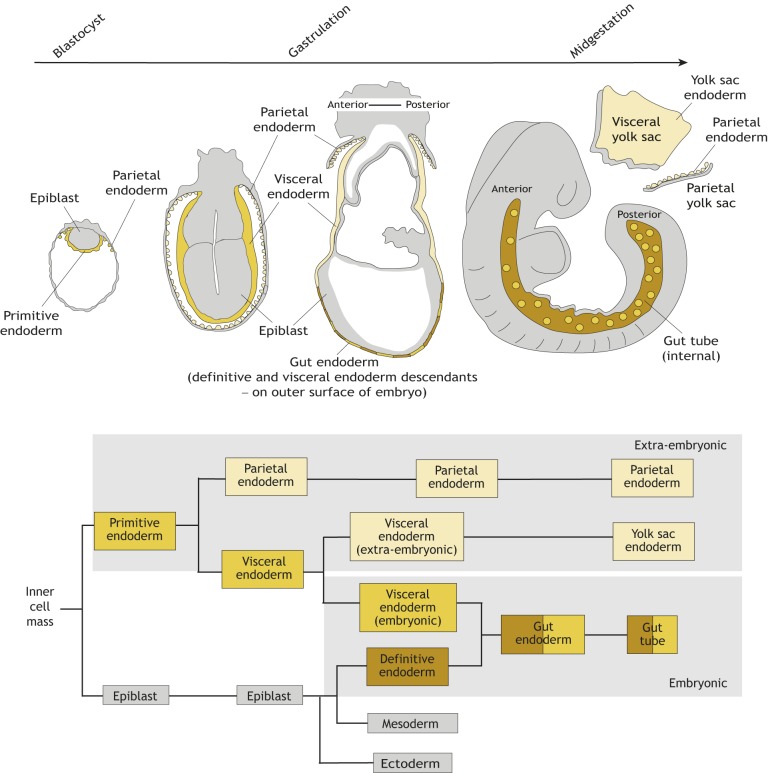
**The endoderm of the mouse embryo arises from two sources with distinct developmental origins.** Schematic overview (top) and lineage tree (bottom) depicting the development of endodermal tissues in mice, from the blastocyst stage to the midgestation embryo. The gut endoderm forms on the surface of the embryo at gastrulation where definitive endoderm (derived from the epiblast; brown) cells intercalate (egress; Schöck and Perrimon, 2002) into the overlying visceral endoderm (derived from the primitive endoderm, yellow). The gut endoderm then becomes internalized and forms the gut tube and will give to the epithelial lining of all endodermal tissues in the adult organism. The gut tube therefore comprises cells of two different origins: extra-embryonic (beige) and embryonic (yellow). Parietal and yolk sac endoderm (beige), which are also derived from primitive endoderm in the blastocyst, solely give rise to extra-embryonic structures.

Endoderm morphogenesis in mammals displays a number of unique features not observed in other organisms. Mammalian embryonic development is unique in that the embryo predominantly develops *in utero* and comprises both the embryo proper and its associated extra-embryonic tissues, which are essential for embryo development, but dispensable for adult life. Endoderm cells are found in both the embryo-proper and extra-embryonic tissues. Indeed, cells with an endodermal identity arise at two distinct times during mammalian development. So-called extra-embryonic (or primitive) endoderm arises in the preimplantation (namely, before the embryo implants into, and makes a connection with, the maternal uterus) embryo from inner cell mass cells (Chazaud and Yamanaka, 2016; Schrode et al., 2013). Thereafter, embryonic (or definitive) endoderm is specified from the pluripotent epiblast at gastrulation (Fig. 2). Primitive endoderm cells predominantly give rise to the endoderm layers of the visceral and parietal yolk sacs, which are two extra-embryonic membranes crucial for the transport of nutrients to the developing embryo and, in egg-laying amniotes, the handling of metabolic waste (Sheng and Foley, 2012). By contrast, definitive endoderm gives rise to the gut tube, which runs the anterior-posterior (or mouth-to-anus) length of the embryo and from which endodermal organs will bud off.

Although visceral endoderm is chiefly responsible for nutrient transport, it also provides important signals directing the establishment of the anterior-posterior axis of the mouse embryo. This results in the posterior localization of the primitive streak, the morphological site where epiblast cells lose pluripotency and undergo an EMT as they acquire mesoderm and endoderm identities, heralding the start of gastrulation (Arnold and Robertson, 2009; Beddington and Robertson, 1999; Lewis and Tam, 2006; Rivera-Pérez and Hadjantonakis, 2014; Tam et al., 2007).

Notably, the segregation of embryonic versus extra-embryonic endoderm lineages is not as strict as initially believed, and we now know that cells derived from the (extra-embryonic) visceral endoderm lying adjacent to the developing epiblast contribute cellular descendants to the embryonic gut tube (Kwon et al., 2008; Nowotschin et al., 2019). This mixing of these two (embryonic and extra-embryonic) endodermal populations occurs when definitive endoderm cells intercalate (see Glossary, Box 1) with visceral endoderm cells, and collectively give rise to the embryonic gut endoderm (we use the term ‘gut’ endoderm to denote the tissue comprising two populations of endoderm cells, definitive and visceral) and resulting gut tube (Kwon et al., 2008; Tremblay, 2010; Viotti et al., 2014a). Notably, descendants of extra-embryonic (visceral) endoderm comprise ∼15% of the gut tube at midgestation (Nowotschin et al., 2019), and it remains to be determined whether they also contribute cellular descendants to endodermal organs in adults.

Although the endoderm of the embryo-proper of mouse, and possibly other mammals, arises from two populations of cells specified at distinct times during development (Chan et al., 2019; Nowotschin et al., 2019; Pijuan-Sala et al., 2019), these cells do share many similarities with the populations of endoderm cells found in other organisms. To begin to make cross-organismal comparisons, however, it is important to ground our understanding of how the endoderm lineage is considered in different model systems. The endoderm has been classically defined as the innermost tissue present throughout the bodies of metazoans – bilaterians and cnidarians. It is often referred to as the gut tube of the developing embryo. However, species-specific variations do exist in what can be considered an intrinsically conserved body plan. For example, although the endoderm forms the midgut in *Drosophila melanogaster*, the most anterior and posterior portions of the gut tube (representing the fore- and hindgut) are ectodermally derived, and in the nematode worm *Caenorhabditis elegans* parts of the foregut are derived from both ectoderm and mesoderm (Maduro and Rothman, 2002). In contrast, the mammalian fore-, mid- and hindgut are all endodermally derived, with the anterior ectoderm-endoderm boundary residing at the back of the mouth (Soukup et al., 2013), and the posterior ectoderm-endoderm boundary dividing the upper two-thirds and lower third of the anal canal (Martín-Durán and Hejnol, 2015). Another way that endoderm cells can be characterized is as typically comprising an epithelial barrier, often exhibiting specialized secretory or absorptive functions. However, there are also significant species-specific differences or adaptations in these functions, with endodermal tissues in *Drosophila* and *C. elegans* dedicated to digestion, whereas those in chordates also give rise to organs required for gas absorption. These species-dependent variations in endoderm contributions and functions can often complicate cross-organism comparisons of endoderm development.

## Mesendoderm – a bipotential progenitor or a potential misnomer?

Another potential obstacle to making cross-organism comparisons is the word ‘mesendoderm’, which is generally used to describe bipotential precursors found both prior to and during gastrulation in several model systems. Mesendodermal cells have the potential to give rise to both mesoderm and endoderm cells (Tada et al., 2005). For example, in the early *C. elegans* blastula, the so-called EMS cell divides to give rise to both the E blastomere, from which the entire endoderm lineage derives, and the MS blastomere, descendants of which contribute to body wall muscle and the posterior half of the pharynx (Leung et al., 1999). Thus, in *C. elegans*, the EMS cell can be considered a mesendoderm cell. The presence of bipotent precursors of mesoderm and endoderm has also been elegantly demonstrated in a series of lineage-tracing experiments in zebrafish, which identified a small number of single cells giving rise to both mesoderm and endoderm (Warga and Nusslein-Volhard, 1999). Furthermore, grafting experiments in chicks showed that both anterior and posterior primitive streak cells, which include endoderm/mesoderm and mesoderm precursors, respectively, can change their fates when placed in a new environment, suggesting bipotent mesendodermal cells (Hatada and Stern, 1994; Kimura et al., 2006).

Even though formation of endoderm and mesoderm is linked throughout evolution, and in mammals there is no proper endoderm formation without proper formation of mesoderm, the existence of bipotent mesendodermal precursors in mammals has been debated. Cells with mesendoderm potential have been proposed to arise in mouse and human pluripotent stem cell differentiation protocols (Hart et al., 2002; Kubo et al., 2004), and clonal analyses have been used to demonstrate the bipotentiality of single differentiated cells *in vitro* (Tada et al., 2005). Evidence for bipotent mesoderm cells *in vivo* was suggested by the finding of descendants in endoderm and mesoderm lineages after the labelling of cells in the anterior primitive streak (Lawson et al., 1991; Lawson 1999). These observations were confirmed in the chick embryo (Hatada and Stern, 1994; Kimura et al., 2006). However, cells with mesodermal potential occupy a broader region within the mouse epiblast, and how a potential *in vitro* mesendodermal cell relates to *in vivo* events, where the time and position of a cell exiting the primitive streak dictates its fate, remain open questions.

Recent cell lineage-tracing experiments using a Foxa2-Cre mouse line have shown that cells having expressed Foxa2, a marker which when expressed in combination with brachyury (T) marks axial mesoderm, whereas in combination with Sox17 marks definitive endoderm, can also contribute to cardiac mesoderm (Bardot et al., 2017). One could therefore speculate that, at least in the mouse, Foxa2-expressing cells might have the potential to become both, endoderm or mesoderm, and only upon activation of Sox17 expression will they commit to endoderm. Moreover, Sox17 expression may require inductive signals from surrounding cells, for example the visceral endoderm. If not exposed to inductive signals, cells might acquire a mesodermal fate, and consequently fail to intercalate into the overlying visceral endoderm.

A feature often used to define a cell as mesendodermal is the co-expression of markers for both endoderm and mesoderm. For example, in early zebrafish embryos, Gata5 and No tail (Ntl; also known as T-box transcription factor T or brachyury in amniotes), are markers for endoderm and mesoderm, respectively, and are co-expressed by cells in the marginal zone, the equatorial area of the blastula that represents the future invaginating endo- and mesoderm (Rodaway et al., 1999). However, although cells co-expressing lineage-specific markers are often referred to as mesendoderm, marker co-expression does not necessarily correlate with developmental bipotential, and even in cases when it does, not all cells exhibiting marker co-expression exhibit a dual fate. For example, in zebrafish, only a small proportion of the cells in the marginal zone divide to contribute to both endoderm and mesoderm lineages (Warga and Nusslein-Volhard, 1999). In mice, Foxa2 and T are markers for endoderm and mesoderm, respectively, and are co-expressed by cells in the primitive streak (Burtscher and Lickert, 2009). Furthermore, many markers classified as being ‘mesoderm’ specific, including T and goosecoid, are in fact expressed within the primitive streak, as well as the nascent mesoderm of the mouse embryo, and so are not specific to a particular lineage per se (Arnold and Robertson, 2009; Chu et al., 2004; Hart et al., 2002). A more extreme case can be found in *Drosophila*, when considering a subpopulation of the anterior endoderm, which is derived from the ventral furrow, a structure that largely forms the mesodermal layer. These cells initially express both Snail and Twist, two transcription factors that are sufficient to drive a mesodermal fate. However, expression of Huckebein, a transcription factor activated downstream of terminal patterning genes, antagonizes the activation of Snail and Twist target genes, and in doing so drives endodermal specification (Reuter and Leptin, 1994). Thus, although co-expression of markers may in some contexts equate with bipotential mesendodermal precursors, such marker co-expressing cells present during normal development predominantly acquire an endodermal fate. Ultimately, the definitive method for assessing the developmental potential of any individual cell is the elucidation of its fate choices during normal development using single cell resolution lineage-tracing approaches.

Interestingly, when considering Cnidarians, the radially symmetric diploblastic (see Glossary, Box 1) sister group of bilaterians, which derive from two germ layers (ectoderm and endoderm), the developmental transcription factor profile of Cnidarian endoderm and its cell functions show striking similarities with that of mesoderm derivatives in bilaterians (Steinmetz et al., 2017). These ‘mesodermal’ gene products in Cnidarians act to regulate cell proliferation, cell motility and adhesion, akin to the role they play within the mesoderm, and during EMT, in higher animals. It is thus likely that, as the mesoderm evolved, genes now ascribed as mesoderm specific became exclusively associated with this particular germ layer (Martindale et al., 2004; Steinmetz et al., 2017; Technau and Scholz, 2003).

## Endoderm internalization and EMT

**Fig. 3.**
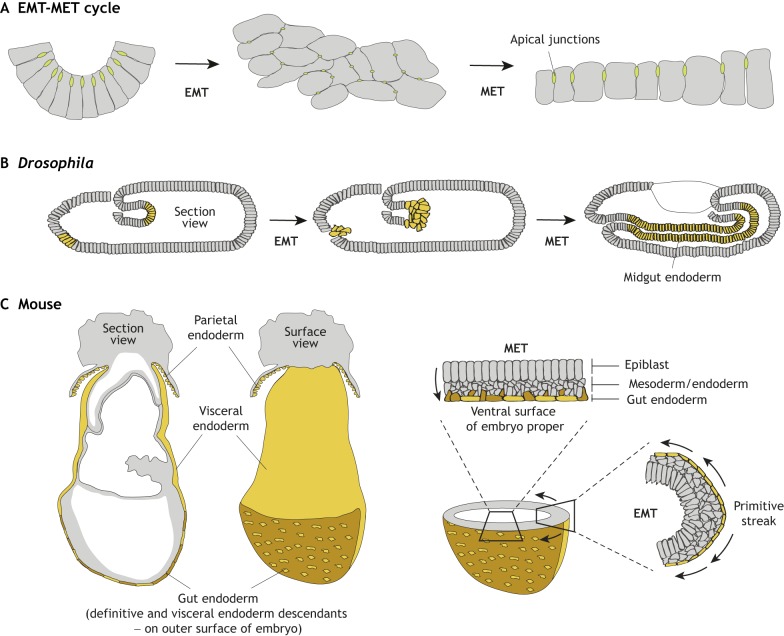
**The EMT-migration/translocation/relocation-MET cycle in *Drosophila* and mouse embryos.** (A) Schematic of cells undergoing an EMT-MET cycle. Cells within an epithelium undergoing EMT lose their apico-basal polarity and loosen their cell-cell junctions (green). After undergoing EMT, cells display a mesenchymal morphology and adopt migratory behaviour. As they rejoin an epithelium and undergo MET, they re-establish cell polarity (by upregulating apico-basal polarized proteins) and reform or reinforce cell-cell junctions. (B) In *Drosophila*, an EMT-MET cycle occurs during midgut formation. Future endoderm cells delaminate from the anterior and posterior poles of the embryo. Cells from both sections migrate and meet to form the midgut section of the gut. Through interactions with the underlying mesoderm, endoderm cells undergo MET and repolarize to form the midgut epithelium. (C) In mice, an EMT-MET cycle occurs during gastrulation. Definitive endoderm cells (brown) undergo EMT when they leave the primitive streak and migrate along the wings of mesoderm. They then undergo MET as they intercalate in the overlying visceral endoderm (yellow) epithelium to form the gut endoderm. Section and surface whole-mount views show the location of the gut endoderm on the surface of the mouse embryo, consisting of definitive endoderm cells and visceral endoderm cells. Cross-sections through the embryo show the position of an EMT at the primitive streak, and an MET during gut endoderm formation.

At gastrulation, massive cell movements reorganize the embryo transforming it into a multi-layered structure. These movements involve coordinated changes in cellular architecture coupled with stereotypical morphogenetic behaviours correlating with an exit from pluripotency and concomitant acquisition of a definitive germ layer fate, coupled with cell internalization. Like the mesoderm, endoderm cells arise at some distance from where they will eventually reside. In *C. elegans*, after specification and internalization, endoderm cells are positioned in the embryo through orientated cell divisions (Leung et al., 1999). However, in most other animals this relocation of endoderm cells within the embryo also relies on endodermal cells undergoing EMT (Fig. 3; see also Box 2), followed by their movement within the embryo, which in some cases has been shown to be an active cell migration process (Campbell and Casanova, 2015; Montero et al., 2005; Wen and Winklbauer, 2017).

Box 2. EMT and cell migration EMT describes a cellular process in which cells with an epithelial phenotype transition to a more mesenchymal state (see also Fig. 3). This transition involves a loss of epithelial characteristics, such as apico-basal polarity, adherens junctions and columnar cellular morphology, and the gain of mesenchymal characteristics, such as increased cellular protrusions and a migratory behaviour (Nakaya and Sheng, 2008; Nieto et al., 2016). EMT is an effective way to bestow on cells a migratory potential. Classically, EMT was considered to be a binary transition from a fully epithelial to a fully mesenchymal cell (Hay, 2005), whereby cells would migrate individually (Nieto, 2011). This would seem incompatible with the behaviour of many endodermal cells types *in vivo*, which often move (or migrate) collectively (Campbell and Casanova, 2015; Dumortier et al., 2012; Montero et al., 2005; Viotti et al., 2014b). However, recent studies have pointed to EMT being a more graded transition whereby cells may adopt a continuum of phenotypes that are bookended by ‘extreme’ epithelial and mesenchymal states, with the more intermediate states being compatible with collective migration of cells (Campbell, 2018; Campbell and Casanova, 2016).

In *Drosophila*, the endoderm is derived from two spatially separated primordia that originate at either end of the embryo: the anterior and posterior midgut rudiments (Fig. 1). These primordia are internalized through invagination (see Glossary, Box 1) at the start of gastrulation. They subsequently undergo collective migration through the embryo until they meet and fuse to form a contiguous tube from the mouth to the anus. Invagination of the posterior midgut has been well studied and shown to be orchestrated by many of the same molecular players known to drive mesoderm invagination (reviewed by Manning and Rogers, 2014). However, whereas invagination and EMT occur consecutively in the *Drosophila* mesoderm, endoderm cells remain epithelial after invagination and only undergo EMT several hours later, through regionally distinct mechanisms. EMT in the posterior midgut is triggered by Serpent, the *Drosophila* orthologue of vertebrate GATA4/5/6 (Gillis et al., 2008), independently of Snail and other so-called ‘EMT-transcription factors’ (Campbell et al., 2011; Lim and Thiery, 2011). Serpent promotes downregulation of junctional E-Cadherin through direct repression of the key apical cell polarity regulator *crumbs* (Campbell et al., 2011). The fact that Serpent disrupts epithelial junctions through repression of apicobasal polarity, rather than of E-Cadherin transcription, appears to be key to enabling the highly ordered collective migration of endoderm cells. As cells initiate migration, E-Cadherin then relocalizes to dynamic punctae on endoderm cell membranes; removing E-Cadherin causes migrating endoderm cells to detach from one another, disrupting their coordinated collective migration (Campbell and Casanova, 2015).

In zebrafish, the endoderm is derived from cells located in the first two cell tiers closest to the margin of the blastoderm (see Glossary, Box 1), a mound of embryonic cells that sits at the animal pole of a large yolk cell (van Boxtel et al., 2018). During gastrulation, endodermal cells internalize individually but in a coordinated manner, in a process that has been termed ‘synchronized ingression’ (Giger and David, 2017; see also Glossary, Box 1). After internalization, endoderm cells migrate anteriorly beneath the epiblast, and eventually come to form a monolayer of cells dispersed with mesoderm precursors. During early somite stages, the endodermal sheet converges on the dorsal midline to form a rod of cells from which organ buds eventually emerge and which later cavitates to form a gut tube (reviewed by Aronson et al., 2014). At early stages of development, when they comprise the outer layer of the embryo, germ layer precursors (including endoderm and mesoderm precursors) form a contiguous epithelioid sheet (Shook and Keller, 2003). These cells lack many epithelial features, but are tightly adherent, whereas after internalization endoderm cells are more mesenchymal in phenotype (Dumortier et al., 2012; Montero et al., 2005), indicating that some degree of EMT must occur. This is supported by a recent finding that the process of endoderm internalization involves the active migration of endoderm cells away from their neighbours, which is triggered by an upregulation of N-Cadherin (Cdh2) in endoderm cells, induced downstream of Nodal, a signal that promotes a mesendoderm fate (Giger and David, 2017). Furthermore, Snail genes are required in a subpopulation of endoderm cells, the axial mesendoderm, for their internalization and this correlates with a temporary downregulation of E-Cadherin (Cdh1) expression (Blanco et al., 2007). GATA transcription factors may also play a role in driving endoderm EMT in zebrafish. Gata5 is expressed in endoderm progenitors prior to gastrulation, and in *faust* mutants, which lack Gata5, the amount of endoderm is reduced (Reiter et al., 1999). In light of recent data from *Drosophila* (Campbell et al., 2011), it would be interesting to revisit the zebrafish *faust* mutant to investigate whether endoderm-EMT and internalization are perturbed. Furthermore, although in *Drosophila* it is clear that Serpent expression and endoderm specification occur a few hours before EMT takes place (Campbell et al., 2011), in zebrafish and amniotes it is not clear whether endoderm specification takes place before, during or after the gastrulation EMT. Moving forward it will be important to determine the timing of GATA factor expression with respect to endoderm lineage specification and EMT.

Less is known about the cellular events that occur during endoderm cell internalization in amniotes in which gastrulation is associated with EMT taking place within the posterior part of the epiblast at the site of the primitive streak (Fig. 3). This event results in the internalization of both nascent mesoderm and endoderm; because their specification occurs in a coordinated manner from a common site of origin, these two lineages are often considered together, with distinctions between them often being blurred. The time and position at which cells exit the primitive streak dictates their fate, with extra-embryonic and cardiac mesoderm exiting first, and paraxial mesoderm and endoderm arising later (Lawson and Pedersen, 1987; Tam and Trainor, 1994). Opposing gradients of signals such as BMP4 and Nodal, as well as the transcription factors T and Foxa2, along the length of the primitive streak have been suggested to respectively direct and reflect cell fate (Burtscher and Lickert, 2009; Morgani et al., 2018), with T^high^ Foxa2^low^ correlating with mesoderm, and T^low^ Foxa2^high^ with definitive endoderm. Nascent mesoderm cells exiting the primitive streak move anteriorly in the space between the adjacent epiblast and visceral (extra-embryonic) endoderm epithelia, exhibiting a collective cell migratory behaviour (Migeotte et al., 2011; Saykali et al., 2019). In mice, nascent definitive endoderm cells first appear about 12 h after the initiation of gastrulation, and they arise from the primitive streak's anterior extremity where the concentration of Nodal (which is present as a gradient along the length of the primitive streak) is highest (Arnold and Robertson, 2009). Live-imaging studies in mice and comparable studies in the chick, whereby endoderm cells can be selectively labelled, have shown that cells specified as definitive endoderm at the primitive streak leave its vicinity, move some distance away, and eventually intercalate into the overlying visceral endoderm thereby forming the gut endoderm (Kimura et al., 2006; Kwon et al., 2008; Viotti et al., 2014b). Thus, the gut endoderm of the mouse embryo comprises cells of two distinct origins: (embryonic) definitive endoderm, and (extra-embryonic) visceral endoderm (Viotti et al., 2014a). It is not currently known whether nascent definitive endodermal cells actively migrate away from the primitive streak, or whether they passively hitchhike a ride on neighbouring migrating mesodermal cells, in a mechanism that might share features with that employed for the internalization of primordial germ cells in *C. elegans* (Chihara and Nance, 2012).

Whereas flies and zebrafish have not yet assembled a cohesive extracellular matrix at the time of endoderm internalization (Latimer and Jessen, 2010; Matsubayashi et al., 2017), in amniotes a basement membrane is localized at the basolateral interface between the epiblast and visceral endoderm tissue layers prior to gastrulation. Breakdown of the basement membrane in the vicinity of the nascent primitive streak, creating a conduit for cell ingression, is a key step in the gastrulation EMT, and has been recognized as the first cell biological sign of gastrulation (Nakaya et al., 2008; Schöck and Perrimon, 2002; Voiculescu et al., 2014; Williams et al., 2012). In chick, basement membrane breakdown coincides with, and is regulated by, destabilization of microtubules at the basal cortex of nascent mesoderm, and presumably nascent endoderm, cells (Nakaya et al., 2008). This is mediated in part through down-regulation of RhoA activity at the basal surface of cells, and likely also through the activation of matrix metalloproteinases (Alev et al., 2010; Nakaya et al., 2013). However, basement membrane breakdown is not sufficient to drive the ingression of nascent mesoderm and endoderm cells. Indeed, in mice that are mutant for *Crb2* (crumbs family member 2), epiblast cells become trapped at the primitive streak despite a clear break in the underlying basement membrane (Ramkumar et al., 2016). Crb2 is required in ingressing cells, which exhibit high levels of apical myosin and segregate from neighbouring cells, for breaking tethers on contracting apical surfaces within the epiblast epithelium as a prerequisite for cell ingression (Ramkumar et al., 2016).

Several recent studies have also provided insights into the roles of E-Cadherin during endoderm morphogenesis in mice. The pluripotent epiblast of the mouse embryo is a columnar epithelium of cells expressing apically and laterally localized E-Cadherin (Lee et al., 2007). As they enter the primitive streak, epiblast cells contract apically and elongate in the apico-basal direction (Ramkumar et al., 2016; Rozbicki et al., 2015; Voiculescu et al., 2014). They concomitantly modulate their levels of E-Cadherin and begin to express N-Cadherin as they ingress (Moly et al., 2016; Viotti et al., 2014b). EMT-associated transcription factors, such as Snail, repress E-Cadherin during gastrulation EMT, and mice that are mutant for Snail genes exhibit a failure of gastrulation EMT (Carver et al., 2001). Interestingly, although nascent mesoderm cells in the mouse embryo appear to lose E-Cadherin, nascent endoderm cells appear to redistribute it, retaining it anisotropically (circumferentially) on their surface (Viotti et al., 2014b), perhaps hinting at their later propensity to repolarize as they intercalate into the adjacent visceral endoderm epithelium, undergoing a reverse EMT, or MET. To complicate matters further, a recent study in the chick revealed that, in addition to expressing E-Cadherin, epiblast cells express the related protein P-Cadherin (Cdh3), and that cells can undergo gastrulation EMT while retaining P-Cadherin in an isotropic distribution on their cell surface (Moly et al., 2016). Indeed, the fact that no antibodies specifically recognizing E-Cadherin are currently available raises the question of whether it is E-Cadherin and/or P-Cadherin that is retained on the surface of nascent endoderm cells, and of the respective roles for different cadherins in the putative EMT-MET cycle of the definitive endoderm.

As in zebrafish (and like Serpent in *Drosophila*), GATA4 and GATA6 are expressed in cells of the primitive endoderm of the mouse blastocyst (Chazaud et al., 2006; Plusa et al., 2008) and in the gastrulating mouse embryo that have downregulated T and migrated away from the primitive streak (Freyer et al., 2015; Simon et al., 2018). Indeed, mouse embryos mutant for *Gata6* exhibit a complete loss of primitive endoderm (Bessonnard et al., 2014; Schrode et al., 2014). However, because GATA4 and GATA6 are co-expressed and are also expressed in both nascent endoderm and cardiac mesoderm, disentangling their unique or redundant lineage-specific roles has been a challenge, and it remains an open question whether GATA factors are required for gastrulation EMT, acquisition of an endoderm identity or endoderm MET in amniotes. The recent development of tools for mesoderm versus endoderm cell lineage-specific gene modulation, as well as reporters for imaging GATA-expressing cells, would merit revisiting GATA mouse mutants to determine more precisely any roles for these evolutionarily conserved factors in the emergent endoderm lineage in the mouse embryo-proper.

Although many of the upstream signals directing cells towards an endoderm identity are conserved between vertebrates, they are not conserved between invertebrates and vertebrates, suggesting that endoderm development may be a very poorly conserved process (reviewed by Zorn and Wells, 2009). However, considering the initial movements of the endoderm in mammals at the start of gastrulation, increasing studies are revealing a number of common characteristics with lower organisms, such as an EMT during the initial internalization, downregulation of E-Cadherin junctions and movement through the embryo. Taken together, this suggests a higher degree of conservation in early endoderm morphogenesis across species than previously suspected.

## Migration of the nascent endoderm

After EMT, endoderm cells in flies, fish and amniotes undergo migration. Live-imaging studies have shown that, despite their mesenchymal appearance, these cells clearly exhibit the coordination and cooperation in migratory behaviour associated with collectively migrating cells (Campbell and Casanova, 2016).

Endoderm cells in *Drosophila* were recently tracked throughout their migration, and parameters relating to their movement during both normal and perturbed development were quantified (Campbell and Casanova, 2015). These studies have shown that endoderm cells in *Drosophila* undergo collective migration, which is mediated by dynamic punctae of E-Cadherin, which are in turn trafficked through the endocytic recycling pathway (Campbell and Casanova, 2015). Similarly, in zebrafish, E-Cadherin expression is maintained in endoderm cells and is actively required for their migration (Montero et al., 2005). Indeed, downregulation of E-Cadherin in zebrafish causes collectively migrating endoderm cells to lose coordinated directionality, and they fail to efficiently migrate towards the animal pole (Dumortier et al., 2012; Montero et al., 2005). Furthermore, Wnt11 has been shown to mediate E-Cadherin dynamics during zebrafish gastrulation through modulation of Rab5, a key component of the endocytic recycling pathway (Ulrich et al., 2005).

In zebrafish, mesoderm and endoderm cells form one contiguous cohesive mass of migrating cells that migrate from the site of ingression towards the animal pole, using the outer ectodermal layer as a substrate. In zebrafish embryos, and also in *Drosophila*, there is no basement membrane separating the two layers; rather, the cells make direct contact with each other, with mesoderm/endoderm cells forming protrusions that contact the neighbouring layer (Clark et al., 2011; Montero et al., 2005; Tepass and Hartenstein, 1994a). The extensive analysis of cell trajectories, morphologies and cell polarization within the prechordal plate, which consists of a subpopulation of mesendoderm cells, revealed that cells show the same behaviour as would be expected if they were to migrate as individuals (Dumortier et al., 2012). However, when single cells are transplanted into host embryos, they fail to migrate towards the animal pole, providing evidence that the migration of these cells is a collective process (Dumortier et al., 2012).

Although the studies discussed above suggest that endoderm cells migrate collectively, they highlight the difficulties that arise when determining whether a group of migrating cells is just a mass of individually moving cells, with the observed collectiveness simply resulting from the fact that all cells respond similarly to the same signals, or a bona fide collective migratory population (as described by Rørth, 2012). The issue of singular or collective, active or passive, migration of cells is particularly relevant when considering the nature of endoderm migration in vertebrates, which has not been elucidated in substantial cellular detail. Indeed, whether endoderm cells in amniotes migrate collectively or individually, actively or passively, remains an open question. Physical forces and tissue tension generated by adjacent cells and the surrounding environment could also contribute to directional movement, as occurs in other morphogenetic processes, such as segregation of germ layer progenitors in zebrafish and directed migration of anterior axial mesendoderm cells in *Xenopus* (Maitre et al., 2012; Weber et al., 2012).

## Endoderm re-epithelialization and MET

**Fig. 4.**
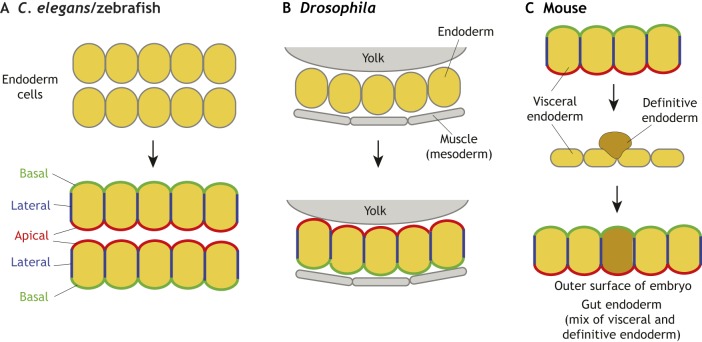
**Endoderm repolarization: the** **different ways cells can re-polarize.** (A) In *C. elegans* and zebrafish, the endoderm comprises a rod of cells that localizes apico-basal polarity proteins at its centre and subsequently generates multiple small lumens, which coalesce into a single one. (B) In *Drosophila*, the endoderm comprises a cup-shaped sheet of cells acquiring apico-basal polarity through cell-cell and cell-basement membrane interactions. (C) In mice, definitive endoderm cells (brown) repolarize as they intercalate into the overlying visceral endoderm (yellow) epithelium. During the intercalation process, visceral endoderm cells relax their apico-basal polarity and cell-cell junctions to facilitate definitive endoderm intercalation. However, once definitive endoderm cells have egressed into the visceral endoderm epithelium, both cell types coordinately repolarize and re-establish cell-cell junctions.

Ultimately, endoderm cells cease moving and must form a functional epithelium. To do this, they need to re-epithelialize by establishing junctions and polarizing. This process is often referred to as MET and/or epithelial differentiation (Fig. 4). However, considering the mesenchymal or partial-mesenchymal state endoderm cells adopt during gastrulation, MET could be considered as a concomitant step in the process of endoderm epithelial differentiation.

In both *C. elegans* and zebrafish, the endoderm initially forms as a solid rod of unpolarized cells, which then undergoes a cord-hollowing process to form a tube. This occurs in the absence of any apoptosis, and instead relies on cell rearrangements and remodelling. During lumen formation, cells first acquire some degree of polarity, and subsequently form multiple small lumina throughout the length of the endoderm. These lumina enlarge and then later coalesce to form a single lumen through the intestinal tube. The emergence of polarity in the *C. elegans* endoderm was first described by Leung et al. Using light and electron microscopy, they showed that the first indication of polarity in intestinal cells occurs after cell divisions have ceased, when cell nuclei move towards the midline and cytoplasmic components move towards the opposite, future basal, surfaces (Leung et al., 1999). At this stage, cells are polarized around the midline, but there is no visible lumen. Soon after cytoplasmic polarization, small and irregular gaps appear between cells, with novel vesicles appearing, which localize to the membranes along the midline. The first regulator of cell polarity to show a polarized localization is PAR-3, which appears in foci that gradually accumulate at the nascent apical surface (Achilleos et al., 2010). PAR-3 foci contain the adherens junction proteins HMR-1 (E-Cadherin), HMP-1 (α-catenin) and HMP-2 (β-catenin). Other proteins required for apico-basal polarity, such as PAR-6 and PKC-3, DLG-1 and AJM-1, which localize to a distinct basal region of mature junctions (Achilleos et al., 2010; Bossinger et al., 2001; Köppen et al., 2001; McMahon et al., 2001), first appear within intestinal epithelial cells after the apical accumulation of PAR-3 and adherens junction proteins is already evident. PAR-3 appears to be the most upstream cue, facilitating polarization by clustering polarity and junction proteins at the cell surface, which then accumulate at apical regions of the cell (Achilleos et al., 2010; Dumortier et al., 2012). As the apical surfaces differentiate, the basement membrane develops at the basal surface of the cells. This mechanism for polarization is distinct from that observed in the ectodermal superficial epidermis, where cells form apical junctions in the absence of PAR-3, and where PAR-6 has a PAR-3-independent role in promoting junction maturation (Achilleos et al., 2010).

Although the overall polarization process of the zebrafish endoderm has been less well studied, the later stages of single lumen formation in the intestinal rod are better understood. Furthermore, although apical membrane expansion is a mechanism used in many ectodermally derived epithelia to drive lumen elongation (reviewed by Andrew and Ewald, 2010), it plays no major role in formation of the gut lumen in zebrafish (Bagnat et al., 2007). Instead, lumen formation initiates with the development of multiple actin-rich foci between cells, followed by the localization of junctional proteins at multiple points within the intestine, and relies at least in part on the activity of aPKC (Horne-Badovinac et al., 2001). Next, these multiple small lumina enlarge through fluid accumulation driven by Claudin 15 and Na^+^/K^+^-ATPase (Bagnat et al., 2007). However, this is not sufficient for a single lumen to form; the remodelling of contacts between adjacent lumina and subsequent lumen fusion is also required. Live-imaging studies combined with genetic analysis indicate that the Hedgehog pathway receptor Smoothened facilitates lumen fusion via Rab11-mediated trafficking and recycling (Alvers et al., 2014). Intriguingly, it was suggested in these studies that Hedgehog signalling from the endoderm may act in the surrounding mesenchyme to mediate some form of signalling or mechanical interactions to regulate lumen fusion, an exciting potential interaction that needs further investigation.

As the *Drosophila* endoderm migrates, it is continually in direct juxtaposition with the so-called visceral mesoderm – the part of the mesoderm that will give rise to the muscles surrounding the gut. The visceral mesoderm plays a key role in both the migration and MET of endoderm cells (Reuter et al., 1993; Tepass and Hartenstein, 1994b). These interactions are known to be mediated by integrins (Devenport and Brown, 2004; Martin-Bermudo et al., 1999) and netrins (Pert et al., 2015), but they likely also involve other signals that are yet to be identified. After migration, once the anterior and posterior endoderm rudiments have met, the now contiguous mass of midgut cells forms a sheet of cells sandwiched between the yolk and the visceral mesoderm, essentially cupping the bottom part of the yolk. This bilayer of tightly adhered endoderm and mesoderm cells then spreads out over the yolk, zipping up at the other surface to envelope the yolk and form a tube. During these morphogenetic movements, midgut cells gradually re-polarize, forming their apical domains on the cell surface facing the yolk, and basal domains at the interface with the visceral mesoderm. Although a highly conserved set of apical polarity proteins, including Crumbs, Stardust (PALS1 in vertebrates) and PALS1-associated tight junction protein, is required for the establishment and maintenance of polarity in the *Drosophila* ectoderm, these are completely dispensable for cell re-polarization in the midgut – they are repressed during endoderm-EMT, and are never re-expressed in the endoderm (Campbell et al., 2011). Instead, embryonic midgut cells appear to rely on lateral-cell interactions mediated by E-Cadherin, and basal-cell interactions requiring integrins, to define their axis of polarity (Tepass and Hartenstein, 1994b).

In mice, definitive endoderm cells repolarize as they intercalate into the visceral endoderm epithelium on the surface of the embryo. As mentioned above, this intercalation results in a gut endoderm formed by cells of two distinct origins: visceral endoderm descendants of the primitive endoderm arising earlier in development in the blastocyst, and definitive endoderm cells arising from the epiblast at gastrulation. As definitive endoderm cells come into contact with the overlying visceral endoderm epithelium, their intercalation (likely constituting an egression event; see Glossary, Box 1; Schöck and Perrimon, 2002) is presumably facilitated by an MET, as they redistribute E-Cadherin apically, and upregulate polarity and cell-cell junctional proteins (Viotti et al., 2014b). To accommodate the intercalation of definitive endoderm cells into the visceral endoderm epithelium, one might speculate that visceral endoderm cells transiently modulate their polarity and cell-cell junctions. Concomitant with the completion of the intercalation process, a new basement membrane is assembled at the interface between the gut endoderm epithelium on the surface of the embryo and the internal mesoderm, in a process that is dependent on the HMG-domain transcription factor Sox17 (Fig. 3), leading to the physical separation of these two adjacent tissue layers (Viotti et al., 2014b). This process is perturbed in mouse embryos mutant for Sox17; indeed, *Sox17* mutants exhibit a widespread failure in definitive endoderm cell intercalation, and their resulting gut endoderm predominantly comprises visceral endoderm descendants (Viotti et al., 2014b). Intercalation is most severely perturbed in lateral and posterior regions of the gut endoderm, perhaps hinting at distinct molecular regulation of anterior endoderm morphogenesis (Kanai-Azuma et al., 2002; Viotti et al., 2014a,b).

Once the gut endoderm has assembled on the ventral surface of the mouse embryo-proper, the foregut invaginates to generate the anterior intestinal portal, while at the posterior a hindgut pocket, the caudal intestinal portal, forms. These two in-pocketings expand and spread posteriorly and anteriorly, respectively, towards the midgut region, where they converge and form a tube as the lateral wall of the midgut folds ventrally, in a process referred to as ventral folding (Tremblay, 2010). The turning of the mouse embryo at midgestation (around embryonic day 9) completes the formation of the gut tube and helps drive its internalization in a process requiring BMP2 signalling (Gavrilov and Lacy, 2013; Kimura et al., 2006; Lewis and Tam, 2006; Madabhushi and Lacy, 2011). The mechanisms of gut tube formation and internalization remain unclear and need further investigation.

When considering different model systems, it is intriguing to note that in *C. elegans*, zebrafish and *Drosophila*, endoderm re-polarization is driven by mechanisms completely distinct from those employed by ectodermally derived epithelia. This was reinforced by a recent study investigating the mechanisms underlying polarization of the endodermally derived midgut stem cells in adult *Drosophila* (Chen et al., 2018)*.* This study found that none of the classic epithelial polarity genes required for polarizing ectodermal cells is required for the apical-basal polarization of adult midgut cells. Furthermore, a germ layer-specific regulation of epithelial polarity has recently been discovered in the diploblastic Cnidarian *Nematostella vectensis* (Salinas-Saavedra et al., 2018)*.* In this context, PAR-3, PAR-6 and aPKC are degraded in the invaginating endomesoderm and are not required for this tissue to form an epithelium. Thus, the difference between endodermal and ectodermal polarity systems may have evolved before the origin of the Bilateria.

## Conclusions and future perspectives

Work in different models has led to the identification of many of the key upstream factors that direct uncommitted embryonic cells into an endoderm identity. However, how this cell fate decision is translated into the stereotypical cell behaviours underlying the early stages of endoderm morphogenesis remains poorly understood. Indeed, we are only now beginning to understand the cell behaviours that characterize the endoderm, from its site of specification to its incorporation into the nascent gut tube. Surprisingly, many of the upstream signals directing cells towards an endoderm identity are not conserved across model organisms. In vertebrates, the TGFβ ligand Nodal is a crucial signal required for the acquisition of an endoderm identity. However, *Drosophila* does not have a Nodal-like ligand, and endoderm identity lies downstream of the activity of the receptor tyrosine kinase Torso, which acts via MAPKK signalling.

The advent of live imaging, gene editing, single cell genomics and optogenetics is opening the door for the interrogation of the dynamic cell behaviours driving endoderm morphogenesis and is suggesting that they might be evolutionarily conserved. These behaviours include a partial-EMT, the collective migration of nascent endoderm cells, a potential reliance on the adjacent mesoderm for this movement to occur, and finally an MET and re-polarization event, which in cases where it has been analysed in some detail, might occur through mechanisms that are distinct from those operating in the ectoderm. However, many open questions remain.
